# Validity evidence for a team‐leading assessment tool in pediatric emergency resuscitations using video review

**DOI:** 10.1002/aet2.10985

**Published:** 2024-04-30

**Authors:** Victoria Hartwell, Katherine Edmunds, Laura Elliott, Brenda Williams, Paul T. Menk, Gary L. Geis

**Affiliations:** ^1^ Division of Emergency Medicine Cincinnati Children's Hospital Medical Center Cincinnati Ohio USA; ^2^ Department of Pediatrics University of Cincinnati College of Medicine Cincinnati Ohio USA; ^3^ Center for Simulation and Research Cincinnati Children's Hospital Medical Center Cincinnati Ohio USA; ^4^ Cincinnati Children's Hospital Medical Center Cincinnati Ohio USA; ^5^ Present address: PM Pediatrics New Hyde Park NY USA; ^6^ Present address: Pediatric Emergency Medicine, Children's Healthcare of Atlanta, and Department of Pediatrics, Emory University School of Medicine Atlanta GA USA

## Abstract

**Background:**

Effective leadership of health care action teams has demonstrated positive influence on team performance and patient care, but there is no consensus on how to assess these skills. We developed a novel team leadership assessment tool for leaders of interprofessional pediatric resuscitation teams and collected validity evidence for this tool using video review.

**Methods:**

This was a prospective cohort study from November 2021 to October 2022. A novel team leadership assessment tool was developed using literature review and local expertise and then piloted and refined using medical simulation. Pediatric emergency medicine (PEM) fellows from a single tertiary care pediatric medical center were enrolled, and videos of one medical resuscitation and one trauma resuscitation were collected per fellow each month. Three reviewers underwent reviewer training and then scored the videos using the assessment tool. Raters provided feedback on feasibility and ease of use using a 5‐point Likert scale. Inter‐rater reliability for the assessment tool using Gwet's agreement coefficient and the association between performance and clinical level of training using generalized linear mixed model were calculated.

**Results:**

Twelve PEM fellows enrolled and 146 videos were reviewed. The inter‐rater reliability for each domain ranged from 0.45 (*p* < 0.0001) to 0.59 (*p* < 0.0001), with the inter‐rater reliability of the total score being 0.49 (*p* < 0.0001). The reviewers’ mean ratings of the elements of the tool were as follows: clarity of the domains (4.6/5), the independence of each domain from each other (3.9/5), the ease of use of the 5‐point Likert scale (4.5/5), the usefulness of the provided examples for each domain (4.6/5), and the ability to assess each domain without having to rewatch (4.5/5). The tool differentiated between levels of clinical training for two of the six domains (*p* < 0.02).

**Conclusions:**

We developed a novel team leadership assessment tool for pediatric resuscitation team leaders that demonstrated moderate inter‐rater reliability. The tool was easy to use and feasible for educators to assess the performance of PEM trainees in complex high‐stakes clinical situations.

## BACKGROUND

Effective leadership of health care action teams, teams that function under complex conditions to perform high‐stakes tasks,^1^ positively influences team performance and improves patient care, including for critical events such as cardiopulmonary resuscitation.^1^, ^2^ As such, graduate medical education and resuscitative care training programs, such as Advanced Trauma Life Support, Advanced Cardiovascular Life Support and Pediatric Advanced Life Support, are now emphasizing the importance of strong leadership skills.^3^, ^4^ Similarly, the Accreditation Council for Graduate Medical Education (ACGME) emphasizes the importance of team leadership in the most recent pediatric emergency medicine (PEM) milestones, which is a framework for the evaluation of the fellow in key areas of physician competence.^5^

While the literature is clear on the importance of the development of team leadership skills in trainees, there is no consensus on how to approach or measure these skills. Current evidence is growing but remains limited.^6^ A systematic review by Rosenman et al.^6^ reviewed 61 assessment tools that evaluated team leadership, of which only 13 assessed leadership as the primary focus. Of note, eight of the tools were derived from the Leadership Behavior Description Questionnaire (LBDQ). The tools that adapted the LBDQ did not provide justification for modifications, and therefore challenges persist in determining if they were accurately measuring the same skills and behaviors as the original scale.^1^ Though team performance assessments with strong validity evidence have been published, these tools typically have been tested on small samples or only have a small portion of the instrument focused on leadership skills, limiting their capacity for meaningful assessment.^7^, ^8^

At this time, there is not a team leadership skill assessment tool for pediatric resuscitation team leaders that has strong validity evidence for actual pediatric patient encounters, which would greatly enhance the training of pediatric resuscitation leaders. Thus, we sought to develop a tool using published literature and local expertise. The intended construct for application of this tool was to assess team leadership during actual patient pediatric emergencies during which care is provided by interprofessional and/or multidisciplinary teams. Our objective was to collect validity evidence at the extrapolation (performance: real life) elemental level of Kane's framework for validity arguments^9^ for this novel assessment tool via video review of real resuscitations. We looked to assess feasibility, ease of use, inter‐rater reliability, and an association between PEM fellow team leadership performance and level of clinical experience.

## METHODS

### Study design and population

This was a prospective study of team leadership performance among PEM fellows based on recorded, real patient resuscitations at a single pediatric tertiary care and Level I trauma institution with a total of 13 PEM fellows. The study was conducted from November 2021 to October 2022 with approval from the institutional review board. All PEM fellows in the institution, excluding those on the study team, were invited to participate.

### Preliminary work

An internally derived assessment tool was developed in 2019 to capture key team leadership behaviors that should routinely and consistently be performed in resuscitative care to improve team‐level situational awareness, teamwork, communication, and management of critically ill and injured children. Three sources were used to identify target behaviors: published literature, local expertise, and an existing video‐based review system of clinical care in our emergency department (ED)'s shock trauma suites (STS).

First, a literature search was performed using PubMed. Articles focused on team leadership, resuscitative care, simulation, and crew resource management were identified, reviewed, and, if applicable, included in the derivation process. Additionally, national meeting abstracts from simulation, resuscitation, and critical care medicine were reviewed for inclusion. Items related to team leadership were identified from existing tools focused on teamwork by one of the authors (G.L.G.) and two educators at our institution's Center for Simulation and Research.

Second, the Center for Simulation and Research has multiple publications surrounding the use of simulation‐based training to improve teamwork and communication.^10^, ^11^, ^12^, ^13^ These studies applied the Team Emergency Assessment Measure (TEAM) and the Mayo High Performance Teamwork Scale (MHPTS) across various settings (in center or lab‐based training, in situ training, and actual patient care) and in various medical and surgical specialties.^14^, ^15^, ^16^ When applying the MHPTS by video review to simulations, we achieved a moderate level of correlation between reviewers.^14^ However, this analysis was based on all 16 items in the tool and not limited to the team leadership items. When applying the TEAM scale by video review in both simulations and real care events, the reviewers were consistent on 96%, again using the entire scale and not limited to the team leadership items.^16^

Finally, the Division of Emergency Medicine's Medical Resuscitation Committee has over 20 years of experience reviewing actual resuscitations for peer review and quality improvement activities. Each bay in the STS is equipped with an audio‐video recording system using a proprietary software program (B‐Line Medical, Washington, DC) that records 24 h per day. All recordings are reviewed daily by a medical resuscitation specialist and maintained in a database. There are written policies for the storage, review, and destruction of these recordings developed with our institution's legal counsel. This infrastructure allowed us to assess whether selected team leadership behaviors could likely be assessed by video review.

The assessment tool was then piloted in a team leadership course for first‐year PEM fellows held in situ in the STS for 3 consecutive years prior to this study. Before each course, the PEM fellowship directors cowatched videos with the first‐year fellows and scored team leadership behaviors using the novel team leadership tool. This was done to prepare the new fellows for the course. Additionally, it allowed the fellowship directors to assess the feasibility and ease of use of the tool as well as provide feedback to the developers on items that needed revision or further explanation. During each course, the tool was applied to in‐person, in situ simulations and provided the format for the debriefing session. After each course, the items selected in the tool were reviewed and iteratively revised by staff from PEM and the Center for Simulation and Research. The current assessment tool has six key leadership skills:

1. Leader is clearly recognized by all team members (Recognized).
2. Leader lets the team know what is expected of them through direction and command (Command).
3. Leader provides an atmosphere of open communication among team members (Open Atmosphere).
4. Leader assures balance between command authority and team member participation (Balance).
5. Leader maintains a global perspective (Global Perspective).
6. Leader communicates clearly with team members (Communicates Clearly).

We borrowed the skills surrounding Command and Global Perspective from TEAM,^7^ changing the wording slightly after feedback from educators and video reviewers. Similarly, we borrowed the skills Recognized and Balance from the MHPTS^8^ with minor changes in wording. The skill Open Atmosphere was inspired by team communication skills described in the literature. However, the literature focused on communication from team members to the leader, and in pilot work we felt the team leader was responsible for creating an atmosphere of communication across all responding team members by removing hierarchies, halo effects, and other obstacles to communication in interdisciplinary care. The skill Communicates Clearly encompasses multiple team leadership qualities that we feel are vital. Initial versions of our tool included the skills of providing team‐level mental model at key moments (e.g., initial mental model, updates, status changes), use of closed‐loop communication, clear reporting of important patient‐level results that impact diagnosis and management, and the development of team‐level situation awareness. These communication elements have all been taught within the ED and by the Center for Simulation since 2005; however, having multiple leader‐specific skills in the tool felt redundant during piloting and was revised into one skill.

Each item is rated on a 5‐point scale of expertise in line with the ACGME Milestones,^5^ defined sequentially as novice, emerging, competency, proficiency, and mastery. To score at the mastery level, a team leader must demonstrate the ability to adapt to changing situations, identify and mitigate potential errors, and recover from situations where team‐based communication has devolved. Examples of high‐level behaviors are listed within the tool to attempt to ensure consistency in application.

The format and proposed use of this tool was based on the Objective Structured Assessment of Debriefing (OSAD) tool derived and validated for simulation‐based debriefing sessions.^17^, ^18^ This 8‐item tool includes scoring in each item ranging from 1 to 5 on a Likert scale. Thus, facilitators can achieve a range of 8–40. The intent of the OSAD is to assess and provide feedback to facilitators on domains in which they struggle during debriefing sessions. Similarly, we developed this team leadership tool with the hope of providing the fellows with feedback on items they struggle with to allow them to focus on improvement in specific areas. Before we can generate validity evidence at Kane's implication level (i.e., demonstrating that improvements in leadership impact care), we must evaluate validity evidence at the previous levels. Our process is mapped on the OSAD's development and validation.

### Outcomes of interest

The primary outcomes of interest were the feasibility, ease of use, and inter‐rater reliability of a novel assessment tool developed to assess team leadership skills of PEM fellows using video review. The secondary outcome was to describe the association between PEM fellow team leadership performance with the level of clinical experience of the PEM fellow, again to collect validity evidence at the extrapolation level of Kane's framework.

### Study procedures

Video recordings from resuscitations were eligible for inclusion if an enrolled PEM fellow was the team leader and the patient was between 0 and 17 years of age. All medical and trauma resuscitations throughout the study were recorded using B‐Line Medical live capture recording software, which provides video and audio recording feedback for four separate views: foot of bed (team view), head of bed (airway view), patient monitor, and situational awareness monitor, which doubles as an endoscopic intubation view when using the Storz C‐MAC system (Karl Storz).

Each month, two eligible videos per PEM fellow were selected for review: a trauma resuscitation and a medical resuscitation. Trauma resuscitations have interprofessional, multidisciplinary teams of 10–14 people, and medical resuscitations have interprofessional teams of eight to 10 people. To remove bias, the first trauma team activation of the month and the first medical team activation of the month per enrolled physician were selected, unless they met exclusion criteria below. Our institution has the following levels of trauma activations, from highest risk of life‐ and/or limb‐threatening injury to lowest: trauma stat, trauma alert, and trauma evaluation. Trauma evaluations are reserved for patients who are hemodynamically stable and have a Glasgow Coma Score ≥ 14, and these were excluded from the study. Medical team resuscitations were only eligible if one of the following conditions were met: airway obstruction, respiratory distress or failure, circulatory shock, altered mental status (including but not limited to status epilepticus), or cardiopulmonary arrest. Selecting trauma stats, trauma alerts, or more critically ill medical patients ensured that interprofessional and/or multidisciplinary care was required and thus was a proxy for requiring team leadership skills. Selecting the initial eligible videos each month prevented needing to randomize or select videos in other ways. Additionally, videos were excluded for the following: (1) the entire resuscitation was not captured on video, (2) the resuscitation lasted less than 5 min (a proxy for low acuity), or (3) if the patient did not require either respiratory support (defined at minimum as application of oxygen) or intravenous access in the resuscitation area to decrease confounding effects of illness/injury severity.^16^ As noted above, the medical resuscitation specialist enters STS encounters daily into a database and has used similar illness severity classification in a previous study^19^; thus, we felt these video eligibility definitions were feasible and able to be reliably applied. We used the same videos and assessment scores to assess the association between PEM fellow team leadership performance with the level of clinical experience of the PEM fellow. The PEM fellows did not receive any feedback on their team leadership performance based on the assessments obtained for this study.

The videos were assessed by three trained reviewers using the Team Leadership Assessment Tool (Appendix S1). To minimize bias, we recruited reviewers who did not work in the STS or work consistently with the PEM fellows. One reviewer is a simulation specialist with a background as a cardiac intensive care unit nurse and does not work with PEM fellows, the second reviewer is a clinical staff pediatrician in the emergency division who does not work with PEM fellows or in the STS, and the third reviewer is a third‐year pediatric resident who only infrequently works with PEM fellows and in the STS.

To train the video review team, patient resuscitation videos obtained prior to enrollment were used in an iterative fashion until the entire review team was consistent in application of the tool; a total of six videos were used. Consistency was defined as scoring within 1 point on the 5‐point scale for each of the six domains. After each training video was scored, the review team met, compared scores, and discussed any inconsistencies. This process continued until the review team achieved consistency in application of the tool. A data dictionary was developed and refined during the reviewer training process (Appendix S2). Once training was completed, two primary reviewers were assigned to each video. Each reviewer was instructed to watch up to the first 20 minutes of each video and then score each video using the team leadership assessment tool. This maximum time was thought to be adequate to evaluate all domains of the team leadership tool. The videos were scored throughout the enrollment period. Each reviewer's 10th video was co‐reviewed by a secondary reviewer (G.L.G.) to prevent scoring “drift.” If there were no discrepancies, the reviewer proceeded with the next 10 videos. Any discrepancies were discussed and agreement achieved before the process was repeated, one video at a time, until consistency was achieved.^20^

Finally, primary reviewers were asked to provide feedback on the assessment tool after reviewing their first, fifth, 20th, and final video. Feedback was measured serially to evaluate for any significant change in the reviewers’ experience with the tool as they became more familiar with it. They were instructed to rate their agreement with the following statements on a 5‐point Likert scale from 1 = strongly disagree to 5 = strongly agree: (1) the clarity of the domains, (2) the independence of each domain from each other, (3) the ease of use of the 5‐point scale, (4) the usefulness of the provided examples for each domain, and (5) the ability to assess each domain without having to rewatch portions of the video. Reviewers were also instructed to provide free‐text comments on the domains, the rating scale, and the ability to apply the tool using video recordings. The data from all surveys were entered into a secured web‐based application (Research Electronic Data Capture [REDCap]).^21^

### Statistical analysis

Gwet's agreement coefficient was used to evaluate inter‐rater reliability. Descriptive summaries on baseline PEM fellow performance measures were reported using mosaic plot. Segmented time series plots were used to depict longitudinal trends of both total score and each individual item. Wilcoxon signed‐rank test was performed to test differences of scores at baseline and follow‐up. We estimated within participants’ correlation and explored longitudinal associations between level of clinical experience and overall scores using generalized linear mixed model. Descriptive statistics were used to describe feasibility and ease of use.

## RESULTS

### Demographics

There were 12 eligible participants; 100% enrolled. Seven (58%) of the participants were female. There were four PEM fellows in each of the three training classes: first year, second year, and third year. All participants had completed at least 4 months of fellowship training prior to enrollment. Two (17%) PEM fellows completed a general emergency medicine residency prior to starting PEM fellowship. All other participants completed a general pediatrics residency.

### Generalizability evidence, feasibility, and ease of use

A total of 161 videos were identified as eligible for inclusion in the study, of which 93 (58%) were for medical resuscitations and 68 (42%) were for trauma resuscitations (Table 1). The proportions of cases are representative of annual trends at our institution. Two (1%) videos were deleted from the server prior to any review, and 14 (9%) videos were deleted prior to review by one of the two reviewers. This resulted in a total of 146 videos that were used to calculate the interrater reliability for the assessment tool. The inter‐rater reliability for each domain of the tool can be seen in Table 2. Overall, the Command domain had the highest level of interrater reliability at 0.59 (95% confidence interval [CI] 0.52–0.67) and the Communicates Clearly domain had the lowest level of inter‐rater reliability at 0.45 (95% CI 0.37–0.52). The inter‐rater reliability of the total score was 0.49 (95% CI 0.42–0.56). A total of 27 videos were co‐reviewed by the secondary reviewer; only one (3.7%) co‐review met the criteria for inconsistency and required a single follow‐up review.

**TABLE 1:** Resuscitation videos eligible for study.

Resuscitation type	Number of videos
Medical	93
Airway obstruction	1
Respiratory distress/failure	51
Circulatory shock	9
Altered mental status (including seizure)	28
Cardiopulmonary arrest	4
Trauma	68
Trauma alert	51
Trauma stat	17
Total	161

**TABLE 2:** Inter‐rater reliability for the Team Leadership Assessment Tool.

Domain	Gwet's AC2	95% CI	*p*‐value
Recognition	0.51	0.43–0.59	<0.001
Command	0.59	0.52–0.67	<0.001
Open atmosphere	0.56	0.48–0.65	<0.001
Balance	0.49	0.41–0.57	<0.001
Communicates Clearly	0.45	0.37–0.52	<0.001
Global Perspective	0.52	0.44–0.60	<0.001
Total score	0.49	0.42–0.56	<0.001

On a 5‐point Likert scale, reviewers rated the clarity of the domains a mean (±SD) of 4.6/5 (±0.36), the independence of each domain from each other at a mean (±SD) of 3.9/5 (±0.17), the ease of use a mean (±SD) of 4.5/5 (±0.2), the helpfulness of the provided examples for each domain at a mean (±SD) of 4.6/5 (±0.17), and the ability to assess each domain without having to rewatch portions of the video at a mean (±SD) of 4.5/5 (±0.34). None of the mean scores changed by more than 0.67 points at any of the timepoints of the study.

In the free‐response section of the reviewer survey, all the reviewers felt that the tool became easier to apply as they reviewed more videos, especially with the use of the supplied data dictionary. One reviewer specifically commented on the tool's 5‐point Likert scale, “I like that there are some ‘in between’ numbers (2 and 4) because it feels like I can give a more accurate rating.” Two of the three reviewers did express challenges with distinguishing between the Command and Balance domains, though one of the reviewers commented that the data dictionary helped to distinguish which behaviors should be attributed to each of the domains, and another reviewer stated that “… after many videos, I had an easier time figuring out which behavior to score in which domain if there was some overlap.” One reviewer commented that team leaders who are quieter were sometimes challenging to score as they inherently score higher on the Open Communication domain based on the definition provided.

### Team leadership performance

**FIGURE 1:**
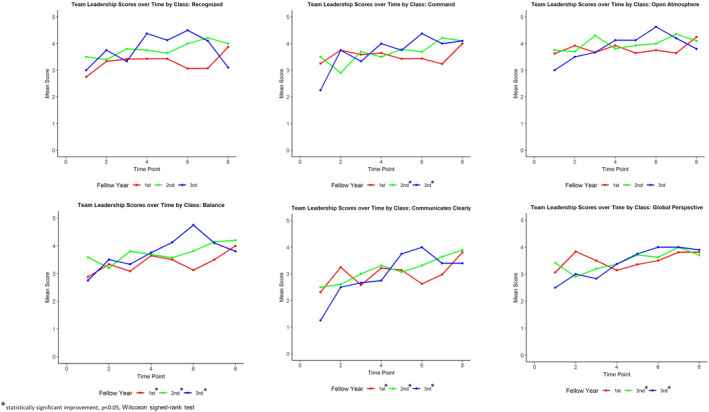
Team leadership performance over time by domain. The graphs depict the changes in performance scores within each domain of the Team Leadership Assessment Tool over the study period, separated by trainee class.

**FIGURE 2:**
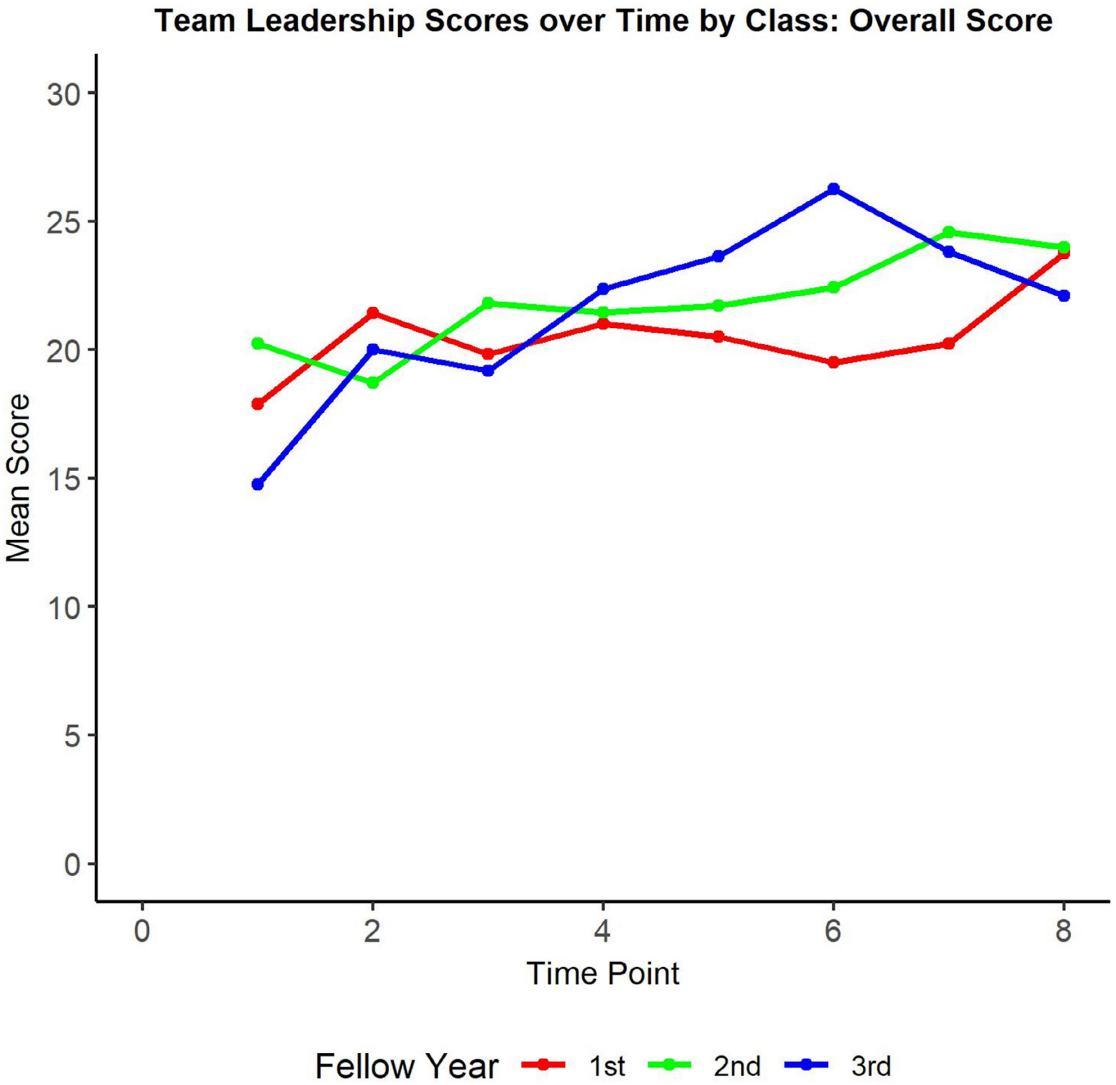
Team leadership performance over time—total score. This graph depicts the change in the overall performance scores as measured by the Team Leadership Assessment Tool over the study period, separated by trainee class.

There was a statistical difference between each training class's scores in the Recognition and Balance domains, with the third‐year PEM fellows scoring the highest for both domains, followed by the second‐year PEM fellows, and then the first‐year PEM fellows. There was no statistical difference between class scores in the other four domains or in the total score (Table 3). Both the third‐ and the second‐year classes had a small but statistically significant improvement in the Global Perspective (0.20 and 0.11, respectively, *p* < 0.05) and Command (0.17 and 0.13, respectively, *p* < 0.05) domains over the course of the study, and all classes demonstrated a small but statistically significant improvement in the Balance domain (0.15, 0.10, and 0.11, respectively, *p* < 0.05), the Communicates Clearly domain (0.24, 0.19, and 0.12, respectively, *p* < 0.05), and the total score (0.88, 0.67, and 0.46, respectively, *p* < 0.05) of the Team Leadership Assessment Tool over the course of the study. The performance of each class over time is shown in Figures 1 and 2.

**TABLE 3:** Team leadership performance by training level.

Domain	First‐year PEM fellows		Second‐year PEM fellows		Third‐year PEM fellows		*p*‐value
	Mean (±SD)	Median (IQR)	Mean (±SD)	Median (IQR)	Mean (±SD)	Median (IQR)	
Recognition	3.3 (±1.12)	3.5 (1–5)	3.76 (±0.89)	4 (1–5)	3.9 (±0.92)	4 (1.5–5)	<0.02
Command	3.49 (±1.01)	3.5 (1–5)	3.65 (±0.84)	4 (2–5)	3.85 (±0.87)	4 (2–5)	0.21
Open Atmosphere	3.79 (±0.79)	4 (1.5–5)	3.93 (±0.75)	4 (2–5)	3.99 (±0.69)	4 (3–5)	0.45
Balance	3.39 (±0.9)	3.5 (1–5)	3.71 (±0.8)	3.75 (2–5)	3.88 (±0.88)	4 (2–5)	<0.02
Communication	2.96 (±1.14)	3 (1–5)	3.17 (±1.07)	3 (1–5)	3.04 (±1.25)	3 (1–5)	0.63
Global Perspective	3.49 (±0.9)	3.5 (1–5)	3.46 (±0.83)	3.5 (1–5)	3.61 (±0.79)	3.5 (1.5–5)	0.66
Total score	20.41 (±5.17)	21 (6.5–30)	21.68 (±4.56)	22 (11–29.5)	22.26 (±4.72)	22.75 (13.5–30)	0.17

Abbreviation: PEM, pediatric emergency medicine.

## DISCUSSION

This study discussed the development of and collection of validity evidence for a novel team leadership assessment tool designed for use in pediatric emergencies with interprofessional and multidisciplinary teams. The Team Leadership Assessment Tool was demonstrated to be feasible and easy to apply using video review and showed moderate interrater reliability. This tool also distinguished between training levels on two of the six domains and was able to demonstrate an improvement in team leadership performance over time for all levels of PEM trainees.

The inter‐rater reliability of each of the six domains and the total score fell within the moderate range of reliability.^22^ This is consistent with what has been published for similar assessment tools. The TEAM assessment tool also demonstrated a moderate inter‐rater reliability; however, most resuscitations used for collecting validity evidence were simulated and only two items on the tool relate to leadership. The authors did not report inter‐rater reliability for individual items, so it is unclear how those specific items performed.^7^ The CALM tool developed by Nadkarni et al.^23^ is another assessment tool designed to provide team leadership feedback for pediatric resuscitation simulations and demonstrates good generalizability and interrater reliability; however, the validation process was limited to 16 video‐recorded simulated scenarios, and a component of the tool measures medical knowledge separate from team leadership behaviors. Rosenman et al.^24^ also published a team leadership assessment tool that demonstrated strong inter‐rater reliability for simulated patient cases, but inter‐rater reliability was much lower for the live encounters with several items showing disagreement. The authors attribute some of the difference in performance to the multiple patient and team confounders and variables that come with live patient care. This heterogeneity will always be a factor when evaluating trainees at the bedside and likely contributed to lower inter‐rater reliability in this study. Despite these challenges, the team leadership assessment tool demonstrated some agreement with each of the domains. One consideration for improving the performance of the tool would be to use raters who are more familiar with the team leadership role and the clinical resuscitation environment. While all the raters in the study completed rater training, none were PEM physicians. Having resuscitation experts as trained raters may improve inter‐rater reliability, as this tool was developed for use by clinical educators to provide feedback to trainees.

Overall, the feedback from the raters on the tool's ease of use and ability to be applied with video review was positive. Having an assessment tool that can be used with video review allows for more opportunities to assess trainees during actual patient encounters with less scheduling limitations. The tool was also able to be used without having to rewatch the video, which may give it the potential to be applied in real time. The raters mentioned that rating got easier over time, and of the 27 co‐reviews performed to evaluate for reviewer drift over the 11‐month study period, only one co‐review was inconsistent and required a follow‐up review. This suggests that there is little rater drift inherent within our training process, which could allow educators to use this tool without having to retrain raters. Reviewers also commented on a few areas that could be clarified. The reviewers expressed occasional difficulty with distinguishing between the Command and Balance domains, and team leaders who had a quieter style of team leading were harder to score on the Open Atmosphere domain. The Balance domain had lower inter‐rater reliability compared to the other domains and may benefit from revisions to allow for better differentiation and clearer guidance on assessing team leaders with a quieter leadership style. Open Atmosphere was also the only domain in which there was not a difference noted between classes nor an improvement seen over time, which further argues for revision for this domain.

The team leadership assessment tool was able to distinguish between training classes in two of the domains. It was also able to demonstrate small but statistically significant improvements in the total score for all classes and in four of the six domains for the second‐ and third‐year classes. This improvement was noted without any of the PEM fellows receiving feedback on their team leadership performance based on their scores. The medians are all less than 0.6 points from the mean, and the interquartile ranges for the domains are not clustered. This suggests that the tool can distinguish levels of performance and could be used by program directors to monitor the development of trainees’ leadership skills over the course of their training or to assess PEM fellows on the Interprofessional and Team Communication Milestone from the ACGME.^5^ The first‐year class completed the simulation‐based team leadership training course just prior to enrollment in this study, and this may have contributed to not capturing any improvement that occurred during the course.

The validity evidence collected for this tool is based on the construct of pediatric resuscitations conducted by interdisciplinary teams at a large academic center. However, the leadership skills being evaluated are not specific or inherent to the pediatric population and we believe would be generalizable to other emergency‐ or critical care–based settings where resuscitations are performed by interdisciplinary teams.

Future directions could include editing the tool and retesting it to better differentiate between the domains. Another consideration would be evaluating the tool's performance in real‐time at the bedside. Since the reviews were all completed using video review, there is not the capability to provide real‐time feedback on team leadership performance. Using the tool at the bedside would allow trainees to be able to apply feedback immediately. Also, this tool was evaluated using only trainee videos, and evaluating the tool's performance with more experienced physicians could provide a method to objectively evaluate ongoing clinical performance.

## LIMITATIONS

There are several limitations to this study. All fellows were debriefed using this assessment tool in their first year of fellowship, which was 0–2 years prior to the study depending on each fellows’ class. Although they did not formally use the assessment tool beyond the initial team leadership course, this does have the potential to influence how they performed. Specifically, we did not query the fellows on whether they continued to use this tool as a reference after they participated in the leadership course. Using actual patient encounters does not allow for controlling factors that may influence team leadership performance such as time of day, experience level of other staff members, patient acuity, or status of the ED. This limits the amount of validity evidence we were able to collect when compared to simulated cases where more variables are controllable. Also, while our raters completed reviewer training, they do not practice in the STS, which may impact how the team leaders were scored. Lastly, the cohort of PEM fellows was small, with only four fellows per class, which limits the interpretation of any differences in performance.

## CONCLUSIONS

We developed and collected validity evidence at the extrapolation level based on Kane's framework for a novel team leadership assessment tool for pediatric emergency medicine fellow trainees during real resuscitative care. This tool allows educators to assess pediatric emergency medicine trainees in real, complex high‐stakes clinical situations, to provide specific and actionable feedback, and to monitor learner growth over time.

## AUTHOR CONTRIBUTIONS

The authors’ contributions are as follows: study concept and design (Victoria Hartwell, Katherine Edmunds, Gary L. Geis), acquisition of the data (Victoria Hartwell, Laura Elliott, Brenda Williams, Paul T. Menk), analysis and interpretation of the data (Victoria Hartwell, Katherine Edmunds, Laura Elliott, Brenda Williams, Paul T. Menk, Gary L. Geis), drafting of the manuscript (Victoria Hartwell, Gary L. Geis), critical revision of the manuscript (Victoria Hartwell, Katherine Edmunds, Laura Elliott, Brenda Williams, Paul T. Menk, Gary L. Geis), statistical expertise (Victoria Hartwell, Gary L. Geis), and acquisition of funding (N/A).

## FUNDING INFORMATION

This work was supported by internal funds from Cincinnati Children's Hospital Medical Center, Division of Emergency Medicine, Department of Pediatrics.

## CONFLICT OF INTEREST STATEMENT

The authors declare no conflicts of interest.

## Supporting information


Appendix S1.

Appendix S2.
