# Supplementary material for: Validity evidence for a team‐leading assessment tool in pediatric emergency resuscitations using video review
Source: AEM Educ Train. 2024 Apr 30;8(3):e10985. doi: 10.1002/aet2.10985 (PMC11058601; doi:10.1002/aet2.10985)
Supplement: Supplementary file 1 — Appendix S1. [file AET2-8-e10985-s001.docx]

**Appendix 1: Novel Assessment Tool to Assess Team Leadership Rating During Interprofessional Resuscitative Care**

Date of resuscitation: ____________

Time of resuscitation: ____________

STS of resuscitation (circle one): 1 2 3 4 5

Team leader of resuscitation: ____________

Reviewer of video: ____________

Date of review: ____________

| **Team Leadership Skills** | **Example(s)** | **Rating***  (circle one) | **Notes/Comments:** |
| --- | --- | --- | --- |
| Leader is clearly recognized by all team members | *There is one identified leader through the majority of the resuscitation* | 1 2 3 4 5 |  |
| Leader lets the team know what is expected of them through direction and command | *Leader assigns roles and responsibilities; when appropriate, roles are shifted to address urgent or emergent events* | 1 2 3 4 5 |  |
| Leader provides an atmosphere of open communication amongst team members | *Team members are willing to speak up/contribute to situation awareness; team members are willing to ask clarifying questions to team leader* | 1 2 3 4 5 |  |
| Leader assures balance between command authority and team member participation | *Noise level is appropriate; when needed, leader is able to bring team back together by performing a “step back”* | 1 2 3 4 5 |  |
| Leader maintains a global perspective | *Leader remains “hands off” by delegating tasks to other team members* | 1 2 3 4 5 |  |
| Leader communicates clearly with team members | *Leader performs mental models, at appropriate times, to develop team-level situation awareness; i.e. development of the shared mental model* | 1 2 3 4 5 |  |

*Definitions of ratings:

1. Is considered a **novice** in this skill, with areas that are identified by rater/reviewer that leader needs improvement supported by direct examples
2. Demonstrates **emerging** skill, but is inconsistent, deferential or not confident at times during the resuscitation
3. Demonstrates **competency** in this skill while leading teams in the care of moderately ill/injured patients or more straight-forward patients
4. Demonstrates **proficiency** in this skill while leading teams in the care of severely ill/injured, complicated or challenging patients
5. Demonstrates **mastery** consistently in this skill, including the ability to adapt to changing situations, identify and mitigate potential errors, and recover from situations where team-based communication has devolved
