# Supplementary material for: Validity evidence for a team‐leading assessment tool in pediatric emergency resuscitations using video review
Source: AEM Educ Train. 2024 Apr 30;8(3):e10985. doi: 10.1002/aet2.10985 (PMC11058601; doi:10.1002/aet2.10985)
Supplement: Supplementary file 2 — Appendix S2. [file AET2-8-e10985-s002.docx]

**Data Dictionary: Novel Assessment Tool to Assess Team Leadership Rating During Interprofessional Resuscitative Care**

| **Team Leadership Skills** | **Example(s)** |
| --- | --- |
| Leader is clearly recognized by all team members | 1 – No one person is identified as the leader, leadership is not present, leadership changes multiple times during resuscitation, or team leader is at foot of the bed (or equivalent position) <30% of the time  3 – A leader is identifiable but changes a few times during resuscitation or another person leads for a short period of time, leader is at foot of bed (or equivalent position) 50-70% of time, some high-level communications are not run through leader  5 – Leader introduces her/himself to team early, there is one identified leader through the entire resuscitation (>90%) and if not leading has handed over that role formally, vast majority of high-level communication is run through leader |
| Leader maintains a global perspective | 1 – Leader moves from foot of bed multiple times, engages her/himself in physical exam, procedures, etc., loses global perspective of patient status  3 – Leader moves from foot of bed a few times, regains global perspective after those movements, at risk of missing pertinent clinical change(s)  5 – Leader remains “hands off” by delegating tasks to other members, is always aware of patient status including identification of clinical change(s) |
| Leader lets the team know what is expected of them through direction and command | 1 – Leader does not provide direction in assessment or management, team has multiple unanswered questions, roles are missing or unclear  3 – Direction from leader is intermittent and sometimes unclear, some role clarification is needed among team, roles are not shifted when needed  5 – Direction from leader is clear and consistent, leader uses assertive statements to prioritize needs, all roles are present and understood; when appropriate, team leader reassigns roles to address urgent/emergent events; leader uses team member’s names and/or roles (i.e. RT, nurse right) when providing direction |
| Leader provides an atmosphere of open communication amongst team members | 1 – Leader does not ask for advice or help, team seems unwilling to approach leader or make suggestions, no questions are asked by team  3 – Some team members are willing to speak up, however side conversations occur of which content sometimes does not seem to reach leader  5 – Team is willing to speak up/contribute to situation awareness; team is willing to ask clarifying questions to leader; leader solicits feedback from team, leader allows team to complete questions and suggestions without cutting them off |
| Leader assures balance between command authority and team member participation | 1 – Leader is only directive, does not take into account team’s concerns or questions even if offered; noise level is high, struggles to get team back together  3 – Leader accepts input from some team members, however seems to be skewed towards physician input; a few times is too passive or too directive  5 – Noise level is low, resuscitation is calm allowing open communication; when needed, leader is able to bring team back together by performing a “step back” |
| Leader communicates clearly with team members | 1 – No mental models are performed, leader only uses side conversations to communicate, no team-level discussion occurs, multiple team members seem confused about patient’s clinical status throughout resuscitation  3 – A few mental models are provided, but not at helpful times (i.e. struggles to get everyone’s attention) or do not include key parts (missing assessment or management), some team members seem confused about the situation late in resuscitation  5 – Leader performs multiple mental models, at appropriate times, to develop team-level situation awareness (shared mental model); gains attention of group during the mental models; communicates results of testing, i.e. CXR, to team; uses close loop communication to assure team the she/he knows tasks are done; team members all seem “on the same page” |
